# SNHG1: Redefining the Landscape of Hepatocellular Carcinoma through Long Noncoding RNAs

**DOI:** 10.3390/biomedicines12081696

**Published:** 2024-07-30

**Authors:** Tiago S. Fonseca, Rui Miguel Martins, Anabela P. Rolo, Carlos M. Palmeira

**Affiliations:** 1 Faculty of Medicine, University of Coimbra, 3000-548 Coimbra, Portugal; rui.martins@uc.pt; 2 Department of Surgery, Portuguese Oncology Institute, 3000-075 Coimbra, Portugal; 3 CNC—Center for Neuroscience and Cell Biology, 3004-504 Coimbra, Portugal; 4 Department of Life Sciences, University of Coimbra, 3000-456 Coimbra, Portugal

**Keywords:** long noncoding RNA, SNHG1, hepatocellular carcinoma, carcinogenesis, diagnosis, prognosis, biomarker

## Abstract

Hepatocellular carcinoma (HCC) represents a global health concern, ranking as the sixth most common malignancy worldwide and the third leading cause of cancer-related mortality. Despite advances in research, the diagnosis and prognosis of such malignancy remain challenging. Alpha-fetoprotein, the current serum biomarker used in the management of HCC, has limited sensitivity and specificity, making early detection and effective management more difficult. Thus, new management approaches in diagnosis and prognosis are needed to improve the outcome and survival of HCC patients. SNHG1 is a long noncoding RNA mainly expressed in the cell and cytoplasm of cells and is consistently upregulated in tissues and cell lines of HCC, where it acts as an important regulator of various processes: modulation of p53 activity, sponging of microRNAs with consequent upregulation of their target mRNAs, regulation of fatty acid, iron and glucose metabolism, and interaction with immune cells. The deregulation of these processes results in abnormal cell division, angiogenesis, and apoptosis, thus promoting various aspects of tumorigenesis, including proliferation, invasion, and migration of cells. Clinically, a higher expression of SNHG1 predicts poorer clinical outcomes by significantly correlating with bigger, less differentiated, and more aggressive tumors, more advanced disease stages, and lower overall survival in HCC patients. This article comprehensively summarizes the current understanding of the multifaceted roles of SNHG1 in the pathogenesis of HCC, while also highlighting its clinicopathological correlations, therefore concluding that it has potential as a biomarker in HCC diagnosis and prognosis.

## 1. Introduction

Hepatocellular carcinoma is the sixth most common malignancy worldwide and the third leading cause of cancer mortality [1]. Since 1980, liver cancer incidence rates have more than tripled while the death rates have more than doubled, with prognosis remaining poor, a 5-year survival rate of around 18%, and high recurrence rates [2]. Major risk factors for this type of malignancy include viral hepatitis (B or C), alcoholic cirrhosis, hemochromatosis, and nonalcoholic steatohepatitis. Although cirrhosis is not present in all HCC cases, it is estimated to be present in over 70% of patients [2].

Currently, alpha-fetoprotein (AFP) is the only serum biomarker used in the management of hepatocellular carcinoma. The diagnostic process involves imaging studies such as ultrasound, CT, or MRI, together with monitoring AFP levels, and, in some centers, liver biopsy [2]. Serum AFP levels can increase the detection rate, which ultimately helps in determining the appropriate treatment modality for a patient. However, elevated levels of AFP can be indicative of not only primary liver cancer but also preneoplastic lesions or conditions, such as chronic active hepatitis and cirrhosis [2].

Effectiveness of using AFP as a screening test for early HCC is uncertain due to its sensitivity ranging from 39% to 64%, specificity ranging from 76% to 91%, and low positive predictive value ranging from 9% to 32% [3]. Moreover, the sensitivity of AFP is subject to various factors, including tumor size. In patients with tumors larger than 3 cm, AFP sensitivity is about 52%, while dropping to 25% if tumors are smaller than 3 cm. The effectiveness of AFP in the early detection of HCC is limited, as most patients with early-stage hepatocellular carcinoma do not exhibit AFP seropositivity. Furthermore, some patients with advanced disease do not display high levels of AFP, which can lead to a delay in diagnosis and worsen the prognosis. The limitations of this biomarker are noteworthy as the early detection of hepatocellular carcinoma plays a crucial role in enhancing patient outcomes and improving survival rates. 

Although tremendous efforts have been made to decipher the molecular mechanisms underlying HCC development and progression, survival rates for most patients are still low, with tumors often being diagnosed at a late stage, leaving healthcare professionals with few to no therapeutic options. Thus, there is still an urgent need to understand more about what happens at the molecular level in HCC, in hopes of identifying new molecular biomarkers that can both help diagnose these lesions at earlier stages and provide insights on how effective therapeutic regimens are. 

With the increasing focus on the involvement of long noncoding RNAs (lncRNAs) in carcinogenesis, a growing number of evidence indicates that certain lncRNAs play significant roles in the progression of HCC and may have substantial predictive value in identifying such lesions. Although the role of other RNA species such as micro-RNAs or small interfering RNAs has been long implicated in the genesis of many malignant neoplasms, the role of lncRNAs is yet not well established. 

LncRNAs encompass a range of RNA species with a size over 200 nucleotides that are not translated into proteins. The majority of lncRNAs are transcribed by RNA polymerase II, and only a minority of lncRNAs are unstable [4]. Unlike other RNA species, lncRNA expression levels are typically lower. However, these species exhibit more pronounced tissue-specific expression patterns, which hints at the crucial roles in cell type-specific processes. In fact, the majority of lncRNAs (~80%) are characterized as tissue-specific, as opposed to mRNAs (~20%) [5]. Since their discovery, evidence has demonstrated that lncRNAs can exhibit varied subcellular localizations, including the nucleus, the cytoplasm, organelles like mitochondria, ribosomes, and exosomes, among others, and that different localization patterns can dictate different functionalities [6]. LncRNAs are implicated in many cellular processes, primarily through the interaction with mRNA, DNA, miRNA, and proteins. Generally, these molecules are involved in the modulation of RNA stability, chromatin organization, and transcription in the nucleus, as well as translation and post-translational modifications in the cytoplasm [6].

So far, it is known that some lncRNAs are closely associated with cancer development, bearing specific roles as promoters or inhibitors of carcinogenesis. Moreover, due to their high tissue specificity, sensitivity, and reliable stability, these markers exhibit significant potential as informative indicators and therapeutic targets in the scope of cancer treatment [7,8]. 

This work focuses on small nucleolar RNA host gene 1 (SNHG1), an lncRNA consistently upregulated in HCC tissues compared to normal liver tissues, which has been identified as an oncogene [8]. This review aims at gathering and exposing the current and most updated evidence on the involvement of SNHG1 in liver carcinogenesis. 

## 2. SNHG1 and Liver Carcinogenesis

### 2.1. The SNHG Family

The SNHG family represents a group of genes that transcribe lncRNAs. These lncRNAs exert their functions in the nucleus and cytoplasm of cells, regulating gene expression at various levels, including chromatin modification, transcription, miRNA sponging, and post-transcriptional processing [9]. Additional to their fundamental roles in cellular biology, SNHG family members have gained attention for their involvement in different diseases, including cancer. Aberrant expression of SNHG lncRNAs has been associated with carcinogenesis at multiple levels. These lncRNAs can act as oncogenes or tumor suppressors, depending on their expression patterns and the cellular context [10]. Although this is an emerging and relatively new area of research, SNHG family members are considered potential biomarkers for diagnosis, prognosis, and therapeutic targets in cancer and other diseases, due to their diverse roles in cellular processes and disease. The evidence so far suggests a strong correlation between SNHG1 and liver carcinogenesis. 

### 2.2. SNHG1

**Figure 1:**
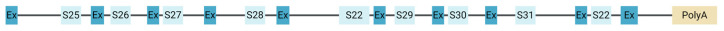
Structure of SNHG1 gene. Dark blue boxes represent exons and light blue boxes represent intronic elements. Adapted from [10] and created using Biorender.com.

Despite research regarding lncRNAs and their role in disease recently acquiring extreme relevance, the first description of the gene organization of SNHG1, also known as U22HG, dates to 1997 [11]. The SNHG1 locus is located on chromosome 11q12.3, occupies less than 5 kb, and produces eight alternatively spliced intronic elements with little potential for protein coding: SNORD22 (126 bp), SNORD25 (69 bp), SNORD26 (75 bp), SNORD27 (72 bp), SNORD28 (75 bp), SNORD29 (65 bp), SNORD30 (70 bp), and SNORD31 (71 bp) [10]. These lncRNAs can all be found in the nucleolus and yield specific functions: SNORD 22, 25, and 29 function in direct site-specific 2′-O-methylation of substrate RNAs, and SNORD 26, 27, 28, 30, and 31 are thought to act as a 2′-O-ribose methylation guide for ribosomal RNA [10]. A schematic representation of the gene structure of SNHG1 can be found in Figure 1. 

**Figure 2:**
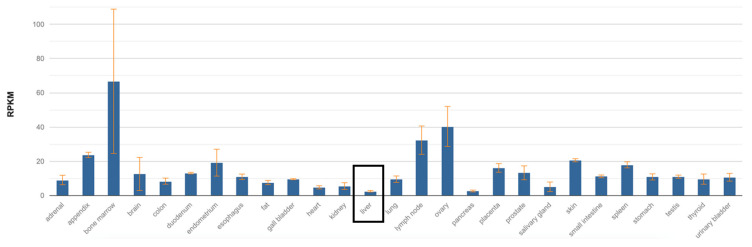
SNHG1 expression in normal tissues. Mean RPKM: 2.542 ± 0.419. Retrieved from RefSeq, 2017, NCBI.

SNHG1 is found in nearly all healthy human tissues, with the liver exhibiting the lowest basal expression, as shown in Figure 2. SNHG1 exists both in the nucleus and cytoplasm of cells, allowing for the diverse functional interactions that this species displays with several other molecules, as elaborated below. 

**Figure 3:**
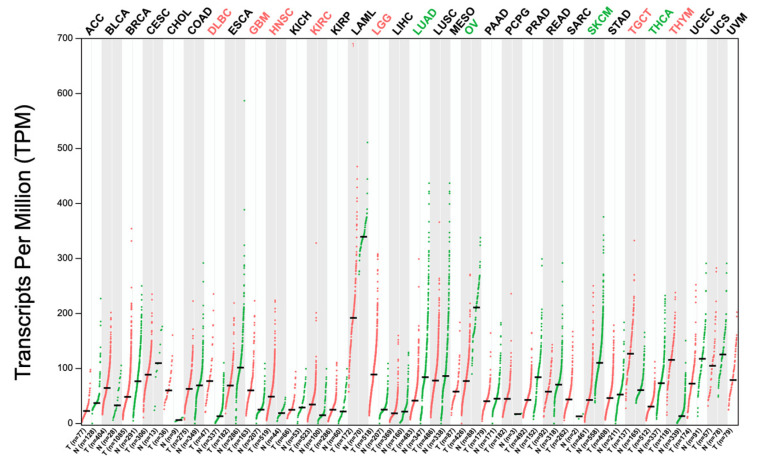
SNHG1 expression across different types of cancer. LIHC: liver hepatocellular carcinoma. Red—normal tissues; Green—cancerous tissues. Retrieved from GEPIA2 database.

**Figure 4:**
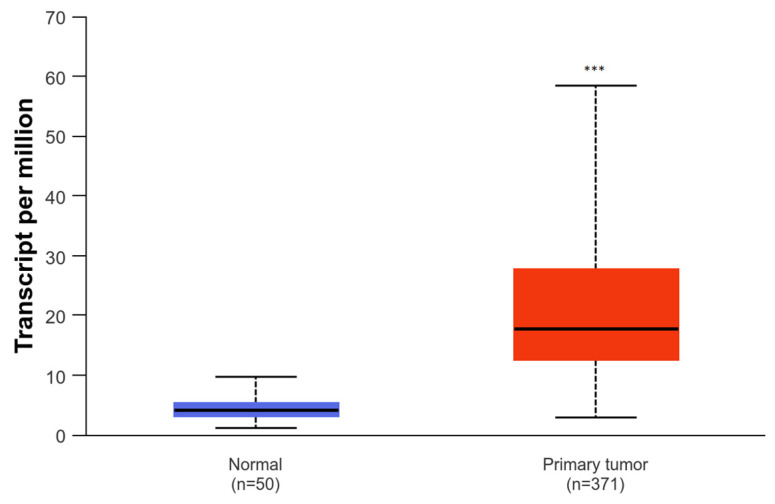
SNHG1 expression in healthy and canceroer tissues. *** *p* < 0.001. Retrieved from TCGA data present on the UALCAN platform.

Based on TCGA data, an in-depth evaluation of the expression of SNHG1 among different cancers shows a significantly increased expression of this lncRNA species in HCC tissues compared to healthy liver tissues, as well as in other forms of cancer (Figure 3 and Figure 4). 

SNHG1 can be a key player in various aspects of tumorigenesis, including cellular proliferation, invasion and migration, apoptosis, angiogenesis, metastasis formation, and drug resistance. In fact, this lncRNA has been demonstrated to promote liver carcinogenesis via four main mechanisms: inhibition of p53 expression and its target genes, modulation of miRNA activity with downstream modulation of target messenger RNAs, modulation of antitumor immune responses by forming a crosstalk with immune cells, and metabolic reprogramming of cancer cells. Moreover, SNHG1 appears to be implicated in treatment resistance in the context of HCC. These topics will be elaborated and discussed in the following sections. 

### 2.3. SNHG1 and p53 in HCC

**Figure 5:**
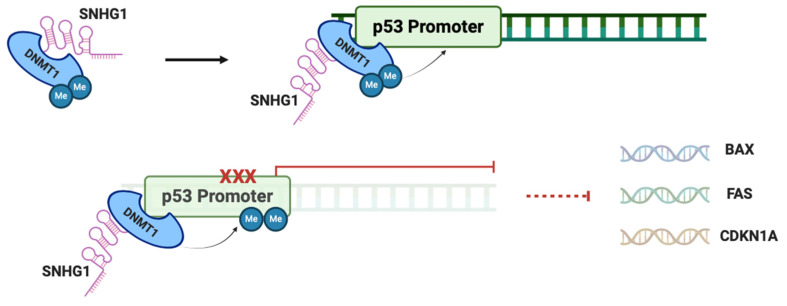
Inhibition of p53 by SNHG1. Through binding to DNMT1, SNHG1 stabilizes the expression of DNMT1, thus promoting the methylation of tumor-suppressor genes like p53. This methylation inhibits p53 and, therefore, its target genes, important for apoptotic signaling and cell cycle arrest, ultimately promoting cell survival and proliferation. Dotted lines indicate omission of steps, displaying the end outcome. Created with Biorender.com.

P53 is an important tumor suppressor protein with a crucial proapoptotic function, being often defined as the “guardian of the genome” [12]. However, upon SNHG1 overexpression in HCC cells, its physiological role becomes compromised. SNHG1 interacts with DNMT1, an important methyltransferase involved in DNA methylation, facilitating its recruitment to the p53 promoter region, thus resulting in increased p53 methylation and consequent inhibition of expression [13]. Through inhibition of p53 there is a downstream inhibition of p53 target genes, such as BAX, FAS, and CDKN1A, important in proapoptotic signaling, cellular response to DNA damage, and cell cycle arrest, respectively [14]. By interfering with these genes, SNHG1 induces cell cycle progression, promotes cellular proliferation, and inhibits apoptosis, resulting in increased invasion ability of HCC cells. This SNHG1-DNMT1 binding mechanism with inactivation of p53, as depicted in Figure 5, has also been shown to promote sepsis-induced myocardial injury by inhibiting Bcl-2 expression, through recruitment of DNMT1 to the Bcl-2 promoter region, contributing to methylation of this gene, to promote the invasion of acute myeloid leukemia cells through recruitment of DNMT1 to the ZCCHC10 promoter region with subsequent inhibitory hypermethylation, and to promote the proliferation of gastric cancer cells [15,16,17]. Altogether, evidence indicates that p53 may play a crucial role as a downstream mediator of SNHG1 effects in promoting HCC development.

### 2.4. SNHG1 and miRNA Activity

LncRNAs can influence tumorigenesis through competitive binding to miRNAs. In fact, lncRNAs are often classified as competitive endogenous RNAs (ceRNAs), and through this mechanism they act as miRNA sponges, competing with mRNA for miRNA binding. miRNAs usually act in the cytoplasm, where they attach to the 3′-end of the target mRNA. This binding either leads to the mRNA’s breakdown or blocks its translation. However, if a ceRNA binds to the miRNA, it prevents the miRNA from attaching to its target mRNA. As a result, the gene that was supposed to be silenced by the miRNA is increasingly expressed because its mRNA is neither degraded nor translationally inhibited [18].

SNHG1 is able to sequester a variety of miRNAs and impair their activity as key regulators in gene expression, which can play important roles in the development and progression of HCC [19]. 

miRNA-195 is part of the miR-15/107 family, which encompasses miRNAs that are inducible and activated in several diseases, including cancer. In HCC, miR-195 has been shown to slow cell cycle progression by blocking G1/S transition, suppress angiogenesis by inhibiting the expression of proangiogenic factors, target signaling proteins like Wnt3a, and dysregulate DNA methylation [20,21,22,23]. miR-195 is a downstream target of SNHG1. Overexpression of SNHG1 directly hampers the expression of miR-195 through direct binding, impairing the normal function of this molecule [24]. Through this mechanism, SNHG1 can also influence the expression of AEG-1. Knockdown of SNHG1 in HCC cells promotes the expression of miR-195 and suppresses the expression of AEG-1, while miR-195 directly suppresses AEG-1, which is a crucial protein for migration and invasion of cancerous cells [24,25]. SNHG1 also promotes the upregulation of PDCD4 and NCAPG, both of which are target genes of miR-195-5p, involved in apoptosis and cell cycle progression, further leading to abnormal proliferation and migration of HCC cells [26,27]. Similar abnormalities are also exhibited in other forms of malignancy, where the interaction of SNHG1 with miR-195-5p results in abnormal proliferation, invasion, and epithelial-mesenchymal transition (EMT) of cells [28]. 

Like miR-195, miR-376a also directly binds to SNHG1. Downregulation of miR-376a in HCC tissues is a direct result of SNHG1 overexpression. However, when SNHG1 is knocked out, this effect is reversed, demonstrating that SNHG1 acts as a negative regulator of miR-376a [29]. Notably, overexpression of miR-376a in HCC cells yields tumor-protective functions by suppressing proliferation and inducing apoptosis [30]. This regulatory effect is partially achieved by the interaction of miR-376a with the FOXK1/Snail axis, which disrupts the expression of proteins associated with migration, apoptosis, and EMT [29]. Consequently, SNHG1 promotes proliferation and invasion of HCC cells while inhibiting apoptosis, by disrupting the normal activity of miR-376a, which is implicated in other forms of malignancy as well [31,32].

SNHG1 is also responsible for inhibiting miR-101-3p in HCC and hepatoma tissues. This miRNA has an extensive molecular network, and is known to inhibit the activity of KIF2C, important for chromosome segregation, SOX9, which acts as an oncogene, SAP30, which regulates histone acetylation, and TKTL1, crucial in aerobic glycolysis and amino acid synthesis [33,34,35,36,37]. By inhibiting miR-101-3p, SNHG1 indirectly upregulates all of these proteins, culminating in HCC proliferation, while being associated with lower overall survival (OS) in HCC patients [33,38]. This SNHG1/miRNA-101-3p axis is also proven to be involved in the progression of osteosarcoma, by inducing higher cell proliferation, migration, and EMT [39].

A similar sponging mechanism is also demonstrated in the interaction between SNHG1 and miR-326 in HCC cells, where overexpression of SNHG1 results in a decreased expression of miR-326 [40]. In HCC, miR-326 is known to inhibit several proteins, like LMNB2, important for the formation of mitotic spindles, FANCE, essential for protection against chromosome breakage, and PKM2, which promotes glycolysis [34,35,36,37,38]. Altogether, the inhibition of miR-326 by SNHG1 promotes the overexpression of these proteins, which in turn promote the proliferation of HCC cells, while predicting a lower OS in HCC patients. Noteworthy, the upregulation of FANCE has been found to also rely on miR-377-3p. SNHG1 inhibits miR-377-3p through direct binding, consequently fostering cell progression and metastasis in HCC [41,42].

Dysregulation of the activity of cyclins in HCC cells seems to be an additionally important mechanism contributing to liver carcinogenesis. By binding to miR-140-5p, SNHG1 releases the inhibitory effect of this miRNA on CDK4, an important cyclin involved in cell cycle progression, eventually promoting the proliferation of HCC cells. SNHG1 is also responsible for the epigenetic silencing of CDKN1A and CDKN2B by interacting with the histone methyltransferase EZH2, thus silencing its target genes, critical in cell cycle arrest [43]. 

Cirrhosis constitutes a major risk factor for HCC and is defined as a premalignant lesion. Bone marrow-derived mesenchymal stem cells (BMSCs) can differentiate into hepatocyte-like cells, which contribute to the highly organized architecture and function of liver tissue, and have been emerging as an effective regenerative therapy for cirrhosis and other kinds of hepatic lesions [44]. A recent study demonstrated that SNHG1 can affect HLC differentiation. The binding of SNHG1 to miR-15a has been demonstrated to result in decreased expression of miR-15a, thereby leading to upregulation of SMURF1 and diminished expression of ATG5 and Wnt5a. SMURF1 has been demonstrated to exert carcinogenic properties in the development of HCC, while Wnt5a has been demonstrated to potentiate HLC differentiation from human MSCs to improve liver function [45,46]. Silencing of SNHG1 significantly ameliorates cirrhosis in livers of mice, while miR-15a mimic treatment in BMSCs restrains cirrhosis in mice, suggesting that SNHG1 silencing alleviates cirrhosis via the miR-15a/SMURF1 axis [47]. 

Finally, through bioinformatic analyses, an additional SNHG1-centered network was studied, suggesting that SNHG1 potentially binds to an additional total of six miRNAs, miR-199a-3p, miR-199a-5p, miR-199b-3p, miR-383-5p, miR-424-5p, and miR-654-3p, leading to the upregulation of six genes, NCAPG, BUB1, TOP2A, CCNA2, KIF11, and CCNB1, in HCC cell lines. All of these are cell-cycle-related genes, contributing to cell cycle progression, and upon SNHG1 knockdown, cell cycle is blocked in the G1 phase [27]. These findings strengthen the notion that the activation of the cell cycle may serve as a primary driving mechanism exerted by SNHG1 in liver carcinogenesis. 

**Figure 6:**
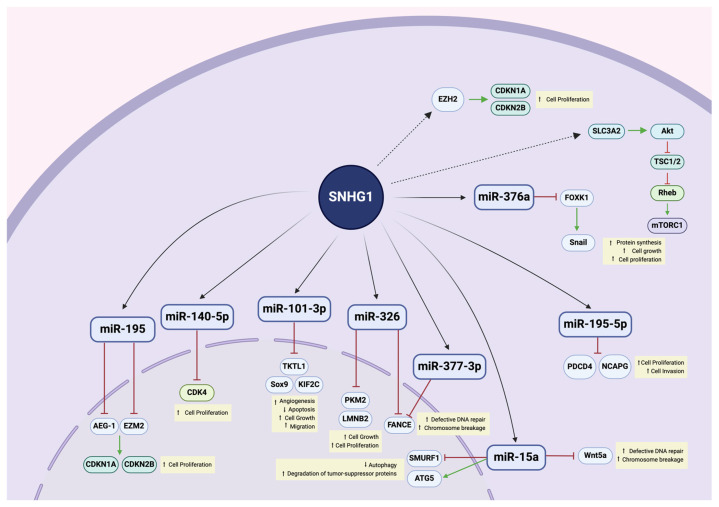
Network of interactions of SNHG1 at the cellular level. SNHG1 interacts with diverse miRNAs through a sponging mechanism, dysregulating downstream expression of important molecules and activation of signaling pathways, leading to increased cellular growth, proliferation and migration, angiogenesis, defective genetic repair, and decreased cellular death. Dotted lines indicate that causality between interaction of SNHG1 with a specific miRNA and the presented outcome has not been proved yet. Green arrows indicate increase in expression and red arrows indicate inhibition of expression. Created using Biorender.com.

In conclusion, the regulatory framework of SNHG1, which encompasses the lncRNA-miRNA-mRNA pathway, contributes to the oncogenic features of this lncRNA. Such interaction network is seen across various types of cancer [48]. Figure 6 summarizes the network of cellular interactions of SNHG1 known to this date. SNHG1 operates on multiple molecular levels, facilitating cell cycle progression through different mechanisms, promoting angiogenesis, EMT, and resistance to genetic defects, while disrupting post-translational processes like methylation and acetylation. 

### 2.5. SNHG1 and Metabolic Reprogramming in HCC

Dysregulation of several metabolic processes, termed “metabolic reprogramming”, has long been defined as a hallmark of cancer, which accelerates cell proliferation and invasion, formation of metastasis, and drug resistance [49]. Through the control of gene expression, protein production, and subsequent enzyme alterations at multiple levels, including transcriptional, translational, and post-translational stages, lncRNAs facilitate metabolic reprogramming observed in all different kinds of cancer, particularly HCC. 

The dysregulation of fatty acids (FAs) metabolism has been identified as a mechanism implied on this metabolic reprogramming in the context of HCC. During tumorigenesis, FAs contribute to the synthesis of cellular membranes and signaling molecules, and changes in FA metabolism are implicated in HCC progression [50]. Upon SNHG1 knockdown, many FA metabolism-related genes, like ACSBG1, become underexpressed in HCC cells. ACSBG1 encodes for the ACSBG1 enzyme, which plays crucial roles in membrane synthesis and several signaling pathways, and its expression appears to be closely related with the expression of SNHG1. Consequently, aberrant expression of SNHG1 can lead to deregulated expression of ACSBG1, thus dysregulating cell signaling and FA production for cell membranes, promoting HCC cell proliferation and migration [51]. 

SNHG1 also appears to be implicated in iron metabolism in HCC. HCC is intimately linked with ferroptosis, a regulated cell death mechanism initiated by lipid peroxidation that is dependent on iron. Ferroptosis is being extensively explored as a strategy to optimize cancer therapy, with research focused on identifying various pharmacological inducers of ferroptosis and potential targets involved in regulating this process [52]. Evidence suggests that HCC cells can inactivate ferroptosis through different mechanisms, and SNHG1 appears to be a key player in achieving this. SNHG1 downregulates the expression of GPX4, NRF2, NCOA4, and CD98, which are implicated in cellular response to oxidative stress and in ferritin metabolism, and upregulates the expression of FANCD2 and G6PD, which are known negative regulators of ferroptosis. Moreover, SNHG1 appears to induce a decrease in the accumulation of intracellular iron and in lipid peroxidation, which, altogether, contribute to the deleterious effects of this lncRNA exerted on ferroptosis [51,53].

SNHG1 is also able to influence glucose metabolism in cancer cells, by accelerating the rate of glucose uptake and production of lactate, known as the Warburg effect. In the context of HCC, SNHG1 interacts with SND1, promoting its binding to SLC7A11, resulting in its upregulation. SLC7A11 represents a key component of the cystine/glutamate antiporter, capable of regulating aerobic glycolysis, and an increase in its expression promotes this metabolic process [54]. SNHG1 also promotes glycolysis in HCC cells by facilitating the expression of PKM2, a kinase with a multitude of interacting molecular partners, which enhances cancer metabolism by promoting the metabolic reliance of cancer cells on glucose [55,56]. 

SNHG1 is integral to the metabolic reprogramming in HCC and its influence extends beyond a single pathway, affecting the metabolism of FA, iron, and glucose, fueling the proliferative and invasive characteristics of tumors and their resistance to cell death. 

### 2.6. SNHG1 and the Immune Landscape of HCC

Research in the field of the immune interactions of lncRNAs is still scarce, but emerging evidence suggests that these RNA species can also influence the tumor microenvironment by influencing recruitment of immune cells. In HCC, SNHG1 can affect immune infiltration by forming a crosstalk with certain groups of cells. Higher SNHG1 expression in HCC is positively associated with a higher infiltration by T and B lymphocytes, while being negatively associated with NK cell and M2 macrophages infiltration [57]. Moreover, a positive correlation has been observed between high SNHG1 expression in HCC cell lines and an augmented expression of important immune-infiltration genes, namely, ICOS, HAVCR2, and PDCD1 [51]. All of these are implied in the regulation of T cell activity, and an aberrant expression can lead to a negative regulation of cytotoxic and regulatory T cell responses, which ultimately promotes the immune escape of HCC cells [58]. Although the exact mechanism through which the increased expression of such genes happens is still unknown, it is reasonable to suspect that it results from the interaction of SNHG1 with specific miRNA species, as has been described above. In fact, SNHG1 is known to promote differentiation and stimulate the function of regulatory T lymphocytes (Tregs) by interacting with miR-448, which can ultimately reduce the cytotoxic activity of lymphocytes against malignant cells, promoting the immune escape of cancerous cells [59]. Moreover, SNHG1 overexpression is known to induce a higher expression of PD-L1 on the surface of malignant cells, leading to the depletion of cytotoxic activity by T cells, while its knockdown increases the CD8+ T cell infiltration in these tumors [60].

The modulation of SNHG1 expression has far-reaching implications in the immune landscape of malignant tumors, namely, HCC, potentially serving as a double-edged sword that not only alters the infiltration and function of various immune cells but also influences the immune escape mechanisms of tumor cells, thereby offering a promising target for therapeutic interventions aimed at enhancing immunogenicity and inhibiting tumor progression.

**Figure 7:**
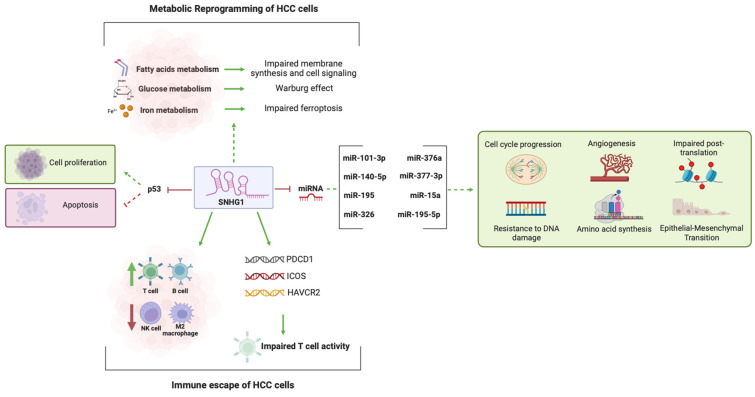
Schematic illustration depicting the molecular and cellular mechanisms through which SNHG1 influences the progression of HCC. Through the inhibition of p53 and its target genes, SNHG1 stimulates cell survival and proliferation, while inhibiting proapoptotic signaling. SNHG1 also impairs the metabolism of FA, iron, and glucose, contributing to the metabolic reprogramming in HCC cells. SNHG1 interacts with a diverse network of miRNAs, inducing cellular processes crucial for carcinogenesis, like inhibition of cell cycle arrest, angiogenesis, EMT, and defective DNA repair and post-translation processes. By influencing the recruitment of specific immune cells and stimulating the expression of important genes, SNHG1 promotes the escape of HCC cells from crucial immune checkpoints. Dotted lines indicate that multiple steps have been omitted, showing only the end outcome. Green arrows indicate the promotion of a process/increase of expression/production and red arrows indicate inhibition of a process/decrease of expression/production. Created with Biorender.com.

The mechanisms outlined in Section 2.3, Section 2.4, Section 2.5, Section 2.6 are summarized and illustrated in Figure 7. 

### 2.7. SNHG1 and Treatment Resistance

SNHG1 can influence the prognosis of HCC by promoting treatment resistance. In HCC, different treatment modalities are employed according to the BCLC stage. A majority of smaller and more localized tumors can be treated by surgical resection, ablation, transarterial chemoembolization, and liver transplant. More advanced BCLC stages are usually managed with systemic therapy, including monoclonal antibodies or kinase inhibitors, like Atezolizumab and Bevacizumab, or Sorafenib and Lenvatinib, respectively [61]. Sorafenib was the initial systemic drug authorized for the management of HCC, and it suppresses the activity of various kinases known to play a role in angiogenesis and tumor cell proliferation, including VEGFR, PDGFR, and Raf [62]. HCC cells that are resistant to sorafenib exhibit abnormally high activation of the Akt pathway, which is critical to cell survival through promotion of cell growth and division, stimulation of angiogenesis, and inhibition of apoptosis, and blocking Akt can potentially reverse this resistance [63,64]. SNHG1 is known to activate Akt by enhancing the transcription of SLC3A2 [65]. Sorafenib triggers the translocation of miR-21 to the nucleus, which subsequently stimulates the expression of SNHG1, leading to the upregulation of SLC3A2 and consequent activation of the Akt signaling pathway [66]. Additionally, SNHG1 contributes to sorafenib resistance by promoting an increase in SLC7A11 expression [54]. This increase can possibly rely on p53, as SLC7A11 is a p53 target and SNHG1 is known to inhibit the activity of p53 [67]. Inhibition of SNHG1 results in the suppression of sorafenib-resistant tumors in vivo, while knockdown of SNHG1 in liver cancer cell lines renders cells more sensitive to sorafenib [54,66]. 

Even though the combination of atezolizumab and bevacizumab has significantly improved the clinical outcome of patients, their response to immunotherapy varies greatly [68]. Recent studies have highlighted the association of a high tumor neoantigen burden (TNB) with improved outcomes in patients treated with immunotherapy [69]. Liver malignant neoplasms with a higher expression of SNHG1 exhibit a significantly lower TNB, as opposed to those with lower expression of SNHG1, which can partially explain why HCC with higher SNHG1 expression can display greater resistance to therapy [57]. 

Additionally, chemosensitivity analyses have demonstrated that lower expression of SNHG1 renders HCC cells more sensitive to several systemic agents, like AKT inhibitor VII, bexarotene, bicalutamide, dasatinib, erlotinib, and gefitinib, while higher expression of SNHG1 can lead to drug resistance [57]. Although doxorubicin has been gradually replaced by new systemic drugs in the treatment of HCC, this compound promotes SNHG1 retention in the nucleus of human cells, by promoting binding of p53 with nucleolin. Consequently, retained SNHG1 traps hnRNPC, preventing this protein from binding and destabilizing p53, leaving it free to exert its crucial regulatory roles upon cellular toxic stress [70].

The relationship between higher SNHG1 expression and treatment resistance in HCC can partially explain why patients respond differently to the same treatment modalities. These newly discovered regulatory networks involving SNHG1 may serve as valuable targets for overcoming resistance to systemic therapy in HCC, and targeting SNHG1 can represent a pivotal strategy in personalizing HCC treatment. Evaluation of the SNHG1 expression profile could potentially help clinicians predict whose patients will respond better, and guide towards more successful and individualized treatment plans that counteract drug resistance and enhance patient prognosis. 

## 3. Clinicopathological Associations between High Expression of SNHG1 and HCC

SNHG1 is reported to be consistently upregulated in HCC tissues and cells, when compared to normal adjacent tissue. A variety of studies have evaluated the correlation between SNHG1 expression and the clinicopathological characteristics of HCC patients, including tumor size, histological grade, presence of lymph node and distant metastases, disease stage (AJCC/BCLC), and AFP levels (Table 1). 

Regarding tumor size, larger tumors significantly correlate with higher expression of SNHG1, as tumors greater than 5 cm exhibit higher expression of SNHG1 as compared to those smaller than 5 cm.

**Figure 8:**
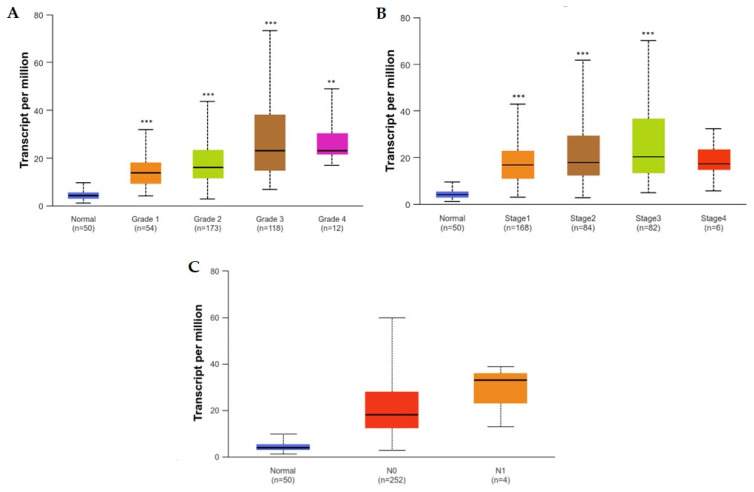
Expression of SNHG1 in HCC based on tumor grade (**A**), on disease staging (**B**), and nodal metastasis status (**C**). ** *p* < 0.01, *** *p* < 0.001, as compared to healthy liver tissues. Tumor grade (1–4) based on WHO classification (well, moderately and poorly differentiated, and undifferentiated), and disease staging (1–4) based on AJCC classification. Retrieved from TCGA data present on [12].

Tumor histological grade also correlates with SNHG1 expression, as more advanced grades/less differentiated tumors display greater expression levels of SNHG1, supporting the notion that increasing levels of SNHG1 might predict more aggressive tumors. In fact, the TCGA data shown in Figure 8A suggest that normal tissue presents with a statistically significant lower SNHG1 expression compared to any histological grade, and that SNHG1 expression is also significantly lower in grade 1 versus grade 3, as well as in grade 2 versus grade 3. Although no significant differences have been found between grades 1/2/3 and grade 4, such might be due to the low number of samples of grade 4 tumors that were included in the analysis, warranting further studies to investigate whether grade 4 tumors exhibit a statistically significant higher expression of SNHG1 compared to lower grades [71]. 

Contradicting data arise for the correlation between the stage of disease and the expression of SNHG1, since, to the best of our knowledge, only 37% of studies did not find a statistically significant correlation between more advanced disease stages and higher expression levels of SNHG1. TCGA data appear to be in line with these studies, where a significantly higher expression of SNHG1 correlates with more advanced disease stages (Figure 8B). Although no significant differences have been found between normal/stages 1/2/3 and stage 4, such might be due to the low numbers of stage 4 cases evaluated. Nonetheless, these incongruences underscore the need for further studies, to investigate whether more advanced stages exhibit statistically significant higher expression of SNHG1 [71].

In the studies presented in Table 1, the presence of lymph node metastasis only significantly correlated with higher SNHG1 expression in one study, whereas the presence of distant metastasis does not appear to correlate with SNHG1 levels. In fact, TCGA data support this notion, where no statistically significant differences appear to exist between N0 and N1 disease (Figure 8C) [71]. 

Regarding AFP expression, it becomes difficult to evaluate if a specific level of AFP correlates, or not, significantly with SNGH1 expression, since all three studies evaluating this parameter have used different cut-off values for AFP (Table 1). However, it appears that AFP levels greater than 200 ng/mL correlate significantly with greater SNHG1 expression.

Interestingly, aberrant expression of SNHG1 has been demonstrated to correlate significantly with a variety of important clinical characteristics in other forms of malignancy, including advanced tumor size, magnitude of vascular invasion, advanced TNM stage, and poorer differentiation [72,73,74,75].

If SNHG1 is to be considered as a factor in the management of HCC, it is crucial to investigate whether this lncRNA can be extracted and quantified from body fluids like blood, and whether the intratumor levels align with measurable plasma levels. While there are limited available data on this matter, evidence indicates that there is a significant decrease in plasma SNHG1 levels following liver surgery in cases of HCC [76]. Moreover, SNHG1 can be detected in significantly higher concentrations in the plasma of patients with other forms of malignancy, which also suggests that this lncRNA can be secreted into the bloodstream [77].

**Table 1:** Clinical significance of high SNHG1 expression in HCC. Blue boxes mean *p* < 0.05. Blank cells mean parameter not evaluated.

Population (n)	Age	Tumor Diameter (cm)	Differentiation	LN Metastasis	Distant Metastasis	TNM Stage	AFP (ng/mL)	Ref.
82	>55 vs. ≤55	<5 vs. ≥5	High/moderate vs. Low	-	-	BCLC A vs. B-C	>20 vs. ≤20	[14]
233	>60 vs. ≤60	-	G1 + G2 vs. G3 + G4	N0 vs. N1	M0 vs. M1	AJCC I-II vs. III-IV	-	[27]
150	≥46 vs. <46	≥7 vs. <7	High/moderate vs. Low	N0 vs. N1/N2	-	AJCC I-II vs. III-IV	-	[29]
358	>60 vs. ≤60	-	G1 + G2 vs. G3 + G4	N0 vs. N1 vs. Nx	M0 vs. M1	AJCC I-II vs. III-IV	≤400 vs. >400	[55]
576	-	-	G1 vs. G2 vs. G3 vs. G4	-	-	AJCC I vs. II vs. III vs. IV	-	[54]
72	<50 vs. ≥50	<5 vs. ≥5	-	-	-	AJCC I-II vs. III-IV	<200 vs. ≥200	[76]
389	>60 vs. ≤60	-	G1 vs. G2 vs. G3 vs. G4	-	-	AJCC I vs. II vs. III vs. IV	-	[78]

AFP—Alpha-fetoprotein; AJCC—American Joint Committee on Cancer; BCLC—Barcelona Clinic Liver Cancer; LN—lymph nodes; TNM—tumor, node, and metastasis staging system.

Additional research examining the presence of lncRNAs in the peripheral blood of cancer patients indicates that the concentration of HULC, a distinct lncRNA, is elevated by 10 to 30 times in the blood of cancer patients compared to that of healthy donors [79]. Similar findings have been observed for other lncRNA species [80]. While there is a lack of additional studies evaluating the presence of SNHG1 in the blood of HCC patients, current evidence seems to corroborate the potential mechanism of extracellular secretion of lncRNAs into the bloodstream. This hints at the possibility that SNHG1 can also be released directly from hepatic tissues into the bloodstream, therefore suggesting that plasma quantification of SNHG1 could serve as a reliable indicator to predict the intra-tumoral production of SNHG1. One additional way of analyzing SNHG1 expression levels in peripheral blood can be through the study of exosomes. Exosomes are extracellular vesicles secreted to different body fluids, including blood, which yield a wide range of functionalities and can carry proteins and genetic material from their cell of origin, signatures which can act as biomarkers in cancer, among other diseases [81]. Studies on HCC have demonstrated that SNHG1 exists in plasma exosomes, while maintaining a high stability, and that extraction of plasma exosomes with further extraction of SNHG1 is a feasible way to quantify SNHG1 levels in the plasma [82,83]. However, solely relying on this approach has obvious limitations, since not all SNHG1 present in HCC cells is found within exosomes. As a result, measuring SNHG1 levels only through exosome analysis may not fully reflect the true expression of this lncRNA within the tumor. 

Extensive research has been conducted with the objective of assessing the diagnostic efficacy of various lncRNAs in HCC. Among these, SNHG1 has emerged as one of the top species exhibiting an exceptional diagnostic performance within a comprehensive panel comprising numerous other species [84]. In one study, SNHG1 yielded a statistically significant area under the curve (AUC) of 0.9312 in distinguishing HCC from healthy samples [33]. In another study, AUC values of SNHG1 were higher than those of AFP (0.92, 95% CI 0.86–0.96 vs. 0.85, 95% CI 0.77–0.90). Sensitivity of SNHG1 appears to be higher than that of AFP (87.3% vs. 64.6%), while specificity is lower (86% vs. 94.6%). Interestingly, the combination of SNHG1 with AFP yields higher values of AUC and sensitivity and a slightly lower value of specificity (0.97, 95% CI 0.92–0.99, 96.4%, 87%), suggesting that incorporating SNHG1 into the diagnostic procedure of HCC can enhance diagnostic accuracy [76]. 

**Figure 9:**
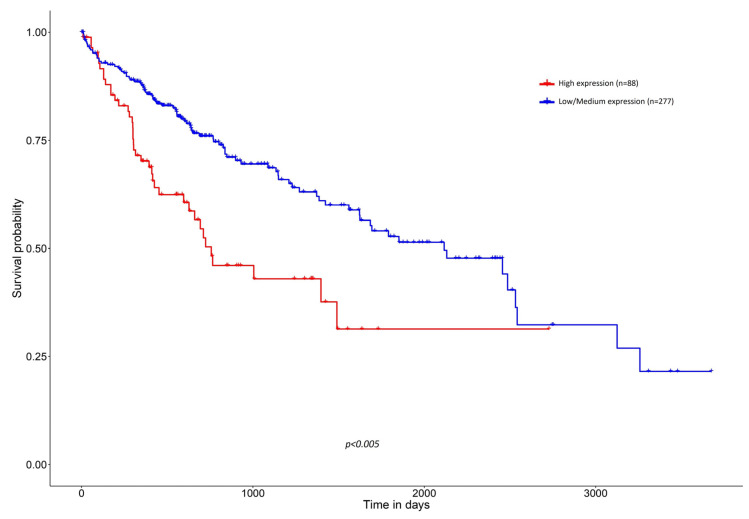
Effect of SNHG1 expression level on HCC patient survival. Retrieved from TCGA database present on [12].

Concerning OS, existing data suggest that high expression of SNHG1 significantly correlates with lower 5-year OS in HCC, strengthening the notion that this lncRNA potentially affects the progression of HCC [14,29,57,78]. In these survival analyses, HCC patients with higher expression of SNHG1 were 35–99% more likely to die when compared to those with lower expression of SNHG1 [27,29,33]. Moreover, data retrieved from TCGA also show a statistically significant lower survival probability in patients with HCC displaying higher SNHG1 expression compared to those whose tumors display a lower expression of SNHG1 (Figure 9). 

The pervasive overexpression of SNHG1 in HCC is significantly associated with critical clinicopathological features and reduced OS, underscoring its potential as a prognostic factor and its influence on the progression of HCC, while suggesting that SNHG1, particularly in combination with AFP, could enhance the diagnostic accuracy for this malignancy.

## 4. Future Directions

The objective of this narrative review was to establish a more solid literary basis for future research projects in the fields of SNHG1 and HCC. As the most updated evidence increasingly underscores the pivotal role of SNHG1 in HCC, there is a growing need for additional research and clinical studies, vital to better ascertain the diagnostic and prognostic potential of this lncRNA, and its involvement in liver carcinogenesis.

From a fundamental research standpoint, it is important to better characterize the subcellular distribution of SNHG1 in HCC and additional human tissues, as its functional interactions with other molecules will rely on their similar subcellular localizations. Bearing this in mind and knowing how SNHG1 interacts with a diverse range of miRNAs in HCC, it is important to continue unveiling new interactions between this lncRNA and other miRNA species to gain a deeper understanding on the complex molecular interactions of SNHG1 and on the effects it can mediate at the level of gene expression and transcriptional and post-translational changes. The same applies to the interactions that SNHG1 has with proteins at the cytoplasmatic level. It seems promising to continue exploring such interactions, which will better elucidate the effect of an aberrant expression of SNHG1 in the vast array of signaling pathways relevant to cancer progression. Moreover, SNHG1 emerges as an important regulator in mediating metabolic reprogramming in HCC cells, modulating the metabolism of FA, iron, and glucose to enhance cancer cell survival in hostile environments. Given the involvement of lncRNAs in many metabolic processes, including also amino acid and glutamine metabolism, it is reasonable to speculate that SNHG1 may also participate in and influence such processes. This suggests unexplored mechanisms through which SNHG1 might contribute to metabolic reprogramming in HCC and hints at new research endeavors in the field of lncRNAs and cancer. 

From a more translational point of view, it is important to better study the extracellular secretion of SNHG1 from HCC cells to plasma, to better understand if SNHG1 can be extracted and quantified from peripheral blood samples and other body fluids, and whether these serum levels correlate well with the intratumoral levels. This would help us to understand if SNHG1 levels in the bloodstream are stable and an accurate representation of the intratumor SNHG1 production. If this proves to be true, then it would be prudent to evaluate the correlation between plasma SNHG1 concentrations and the clinical staging of the disease in HCC patients. Such a systematic evaluation would help us to understand if the expression patterns of SNHG1 can serve as a reliable method to predict with higher accuracy patient outcomes. Furthermore, the importance of SNHG1 in HCC therapies is also promising, underscoring the importance of research to elucidate its intricate mechanisms. As authors navigate the complexities of HCC treatment, SNHG1 emerges as a possible new therapeutic target. Its reduced expression results in a higher TNB and increases the sensitivity of HCC cells to systemic agents, potentially impacting the response of patients to immunotherapy, thus highlighting the key role that SNHG1 can play in shaping the landscape of personalized and targeted interventions for HCC.

Expanding on these questions by performing the mentioned analyses in a larger cohort of HCC patients could help further determine whether SNHG1 expression patterns correlate well with different stages in disease, so that the quantification of serum levels of SNHG1 can be advocated as a reliable prognostic or monitoring method to assess HCC patients. This can point to promising directions for future research endeavors in the field of lncRNA and their utility for cancer management, and ultimately improve early detection, management, and treatment, while achieving better clinical outcomes and higher survival rates for HCC patients.

## 5. Conclusions

SNHG1 is pivotal in HCC pathogenesis, modulating various pathways that compromise cellular proliferation, migration, and invasiveness, alongside the regulation of apoptosis and angiogenesis. Increased expression of SNHG1 has been associated with the development and progression of HCC, and a growing body of research suggests that high expression of this lncRNA is associated with a worse outcome in disease, culminating in more aggressive tumors, treatment resistance, and shorter OS.

It is worth noting that although SNHG1 may be present as an oncogene in other forms of malignancy, its expression levels are highly increased in HCC. Rather than advocating for SNHG1 as a standalone biomarker for HCC management, this review aims to describe the scope of SNHG1’s involvement in HCC and highlight its potential as an additional serum marker to improve detection rates and prognosis prediction. Including SNHG1 in the diagnostic process, for example, through a multi-biomarker panel, could improve outcomes for HCC patients.

As discussed in the text, the management of HCC typically starts with imaging modalities, followed by AFP level assessment and, if necessary, a liver biopsy. Combining SNHG1 with AFP can significantly enhance diagnostic accuracy, yielding higher sensitivity and specificity compared to AFP alone. One interesting note about AFP is that its levels are commonly analyzed in the management of other forms of malignancy, like testicular cancer, yolk sac tumors, or extragonadal germ cell tumors, yet it is still widely advocated as a biomarker for HCC.

SNHG1 serum levels have the potential to provide additional diagnostic and prognostic information, making it valuable in certain clinical contexts, including the earlier stages of HCC. Moreover, unlike AFP, measuring SNHG1 levels appears to improve the prediction of prognosis for HCC patients, providing clinicians with better insights regarding the aggressiveness of the tumor, the survival likelihood, and the selection of appropriate therapies from the outset. By incorporating SNHG1 into existing diagnostic and prognostic frameworks, clinicians can enhance the overall management and treatment outcomes for HCC patients.

While the exact mechanisms by which SNHG1 contributes to the development of HCC remain to be fully elucidated, the breadth of its molecular interactions makes it a central figure in HCC tumorigenesis. Concurrently, its clinicopathological features underscore its potential as a molecular marker for early detection and a prognostic marker for HCC. Accordingly, this underscores the necessity for expanded research and clinical studies. Such endeavors aim to further explore the role of SNHG1 in HCC and to better consolidate its utility in diagnosis, therapeutic strategy, and prognosis, improving survival predictions and redefining treatment efficacy for HCC patients.

## Abbreviations

ACSBG1	Acyl-CoA Synthetase Bubblegum Family Member 1
AEG-1	Astrocyte Elevated Gene-1
AFP	Alpha-Fetoprotein
AJCC	American Joint Committee on Cancer
ATG5	Autophagy related 5
AUC	Area under the curve
BAX	Bcl-2 Associated X-protein
Bcl-2	B-cell lymphoma 2
BCLC	Barcelona Clinic Liver Cancer
BMSCs	Bone marrow stromal cells
BUB1	Budding Uninhibited By Benzimidazole 1
CCNA2	Cyclin A2
CCNB1	Cyclin B1
CD98	Cluster of Differentiation 98
CDKN1A	Cyclin-dependent kinase inhibitor 1A
CDKN2B	Cyclin-dependent kinase inhibitor 2B
CDK4	Cyclin-dependent kinase 4
ceRNA	Competitive endogenous RNA
CT	Computed tomography
DNA	Desoxyribonucleic acid
DNMT1	DNA Methyltransferase 1
EMT	Epithelial-to-mesenchymal transition
EZH2	Histone-lysine N-methyltransferase EZH2
FA	Fatty acid
FANCE	Fanconi Anemia Complementation Group E
FAS	Fas Cell Surface Death Receptor
FOXK1	Forkhead Box K1
GPX4	Glutathione Peroxidase 4
HAVCR2	Hepatitis A Virus Cellular Receptor 2
HCC	Hepatocellular carcinoma
HLC	Hepatocyte-like cell
hnRNCP	Heterogeneous Nuclear Ribonucleoprotein C1/C2
ICOS	Inducible T-Cell Costimulator
KIF11	Kinesin Family Member 11
KIF2C	Kinesin Family Member 2c
LMNB2	Lamin B2
LncRNA	Long noncoding RNA
M2	M2 Macrophage
miRNA	microRNA
MRI	Magnet resonance imaging
mRNA	Messenger RNA
NCOA4	Nuclear Receptor Coactivator 4
NCAPG	Non-SMC Condensin I Complex Subunit G
NK	Natural killer
NRF2	Nuclear Factor Erythroid 2-Related Factor
OS	Overall survival
PDCD1	Programmed Cell Death Protein 1
PDCD4	Programmed Cell Death Protein 4
PDGFR	Platelet-Derived Growth Factor Receptor
PKM2	Pyruvate Kinase M1/2
SAP30	Sin3-Associated Protein 30
SLC3A2	Solute Carrier Family 3 Member 2
SMURF1	Smad Ubiquitination Regulatory Factor 1
SNHG1	Small Nucleolar RNA Host Gene 1
SOX9	SRY-Box Transcription Factor 9
TKTL1	Transketolase-Like-1
TNB	Tumor Neoantigen Burden
TOP2A	DNA Topoisomerase II Alpha
VEGFR	Vascular Endothelial Growth Factor Receptor
Wnt5a	Wnt Family Member 5A
